# When are static and adjustable robust optimization problems with constraint-wise uncertainty equivalent?

**DOI:** 10.1007/s10107-017-1166-z

**Published:** 2017-06-12

**Authors:** Ahmadreza Marandi, Dick den Hertog

**Affiliations:** Tilburg School of Economics and Management, Tilburg University, Tilburg, The Netherlands

**Keywords:** Robust optimization, Adjustable robust optimization, Constraint-wise uncertainty, Hybrid uncertainty, 90C30, 90C46, 90C47, 49K35

## Abstract

Adjustable robust optimization (ARO) generally produces better worst-case solutions than static robust optimization (RO). However, ARO is computationally more difficult than RO. In this paper, we provide conditions under which the worst-case objective values of ARO and RO problems are equal. We prove that when the uncertainty is constraint-wise, the problem is convex with respect to the adjustable variables and concave with respect to the uncertain parameters, the adjustable variables lie in a convex and compact set and the uncertainty set is convex and compact, then robust solutions are also optimal for the corresponding ARO problem. Furthermore, we prove that if some of the uncertain parameters are constraint-wise and the rest are not, then under a similar set of assumptions there is an optimal decision rule for the ARO problem that does not depend on the constraint-wise uncertain parameters. Also, we show for a class of problems that using affine decision rules that depend on all of the uncertain parameters yields the same optimal objective value as when the rules depend solely on the non-constraint-wise uncertain parameters. Finally, we illustrate the usefulness of these results by applying them to convex quadratic and conic quadratic problems.

**Electronic supplementary material:**

The online version of this article (doi:10.1007/s10107-017-1166-z) contains supplementary material, which is available to authorized users.

## Introduction

Many real-life optimization problems have parameters that are not exact. One way to deal with parameter uncertainty is robust optimization (RO), which enforces the constraints to hold for all uncertain parameter values in a user-specified uncertainty region. In RO, all decision variables represent “here and now” decisions, which means they should obtain specific numerical values as a result of the problem being solved before the actual uncertain parameter values “reveal themselves”.

An extension to RO is adjustable robust optimization (ARO) introduced in [4]. In ARO, some of the decision variables are “here and now”, while others represent “wait and see” decisions, which are assigned numerical values once some of the uncertain parameters have become known.

The advantage of using ARO lies in the fact that its worst-case objective value is no worse, and indeed usually better, than the corresponding static RO. In [6] the authors prove for linear problems with linear uncertainty and convex uncertainty set that if the uncertainty is constraint-wise, and under a few more assumptions, RO and ARO have the same optimal objective value. It is shown in [10] that the same result holds even for specific non-constraint-wise uncertainty. The conservativeness of the RO solution for the ARO problem is studied for some classes of problems in [9] and [10].

Solving an ARO problem can be NP-hard even for linear cases [12]. There are accordingly many methods in use for finding a good approximation for an ARO problem. Using affine decision rules, [4], for “wait and see” variables appears to be effective for many ARO problems. For linear ARO problems with fixed recourse, using affine decision rules leads to a robust linear problem that is computationally tractable for many types of uncertainty sets. This is not the case for problems with non-fixed recourse.

An important line of research in ARO is finding classes of problems for which the affine decision rules are optimal. In [8], the authors prove that affine decision rules are optimal for linear ARO problems with right-hand side uncertainty and simplex uncertainty sets. A similar result is proven in [11] for ARO problems with a specific objective function that is convex in the uncertain parameters and adjustable variables, box constraints for the variables and a box uncertainty set. Also, in [16], the optimality of the affine decision rules is proven for unconstrained multi-stage ARO problems under some structural assumptions on the uncertainty set and objective function.

In [7], a bound is derived for the gap between the objective value of the problem that results from using affine decision rules and that of the ARO problem.

Although substituting “wait and see” decision variables with affine functions would appear to be highly effective, the method needs introducing many new variables. This is because for a problem with *n* adjustable variables and *l* uncertain parameters, applying affine decision rules means substituting *n* adjustable variables with $n(l+1)$ non-adjustable variables.

The contribution of this paper is twofold:

1. We prove for convex problems with concave uncertainty, which also satisfy a set of other conditions, that the objective values of the corresponding RO and ARO problems are equal. This is an extension of the result in [6, Proposition 2.1], which is only for problems that are linear in the variables and uncertain parameters.
2. We study uncertain nonlinear problems in which some of the uncertain parameters are constraint-wise and the rest are not. In particular, we prove that for an ARO problem, under a set of conditions similar to the pure constraint-wise cases, there is an optimal decision rule that depends only on the non-constraint-wise uncertain parameters. Moreover, we show that for a specific class of problems, there is an affine decision rule that is only a function of the non-constraint-wise uncertain parameters and that yields the same objective value as using an affine decision rule that is a function of all uncertain parameters.

The first contribution means that for this class of problems, there is no need to solve ARO ones. This has two outstanding merits: first, solving an RO problem is computationally much easier than solving an ARO one; and second, since ARO is an online approach, parts of the solution for a problem can only be implemented once the values of the uncertain parameters are known. The RO approach is an offline one, however, so all preparations for implementing the solution can start immediately upon solving the RO problem (for further discussion about online and offline approaches see [19]).

The merit of the second contribution is that it reduces the number of variables in the problem by using affine decision rules, since we know beforehand that there is an optimal solution for ARO where the coefficients of the constraint-wise uncertain parameters are zero.

In the last part of the paper, we apply our theoretical results to an important class of problems. We show that our contributions are applicable to convex quadratic and/or conic quadratic problems, which can arise in multi-stage portfolio optimization, for example.

We emphasize that the results obtained in this paper concern the worst-case objective value of an ARO problem. We provide conditions under which the optimal RO solutions are also optimal for the ARO problem. However, in such cases, another ARO optimal solution may yield a better average-case objective value [17].

The rest of the paper is organized as follows: Sect. 2 presents our main results. We provide a set of conditions under which constraint-wise RO and ARO problems have the same optimal objective values. Moreover, for problems in which just some of the uncertain parameters are constraint-wise and not all, we show that under a similar set of conditions, there is an optimal decision rule that is independent of the constraint-wise uncertain parameters. In Sect. 3, we apply our results to convex quadratic and conic quadratic problems.

## Main results

In this section, we derive the main results presented in the paper. The section starts by introducing some definitions and preliminaries in Sect. 2.1. In Sect. 2.2, we provide a set of conditions for problems with constraint-wise uncertainty under which adjustable and static robust optimization produces the same optimal values. In Sect. 2.3 we study problems in which only some of the uncertain parameters are constraint-wise and the rest are not.

### Preliminaries

Consider the following uncertain nonlinear optimization problem1where $\zeta \in \mathcal {Z}\subseteq \mathbb {R}^{l}$ is an uncertain parameter and $\mathcal {Z}$ is a nonempty uncertainty set, $x\in \mathcal {X}\subseteq \mathbb {R}^r$ is a non-adjustable variable, and $\mathcal {X}$ is a nonempty set defined by constraints that depend only on $x$, $y\in \mathcal {Y}(x)\subseteq \mathbb {R}^n$ is an adjustable variable and $\mathcal {Y}(x)$ is defined by constraints independent of $\zeta$. Also, we assume that $f(\zeta ,x,y) \text{ and } g_i(\zeta ,x,y)$, $i=1,\ldots ,m$, are continuous.

We can define static and adjustable robust optimization problems corresponding to uncertain problem (1).

#### Definition 1

(*Static robust optimization*) For problem (1), the static robust counterpart (*RC*) is defined by


#### Definition 2

(*Adjustable robust optimization*) For problem (1), there are two different definitions for the adjustable robust counterpart (*ARC*):2and


The equivalence of problems (2) and (*ARC*) is proved in [20]. We denote the objective values of problems (*RC*) and (*ARC*) by *Opt*(*RC*) and *Opt*(*ARC*), respectively.

We extend the definition of (*ARC*) with fixed recourse for a linear problem with linear uncertainty in [4] to the nonlinear case (nonlinear problem with nonlinear uncertainty) in the following definition.

#### Definition 3

(*Fixed recourse problem*) (*ARC*) has fixed recourse when there are continuous functions $\tilde{f},\tilde{g}_i:\mathbb {R}^{n+r}\rightarrow \mathbb {R}$, $\bar{f},\bar{g}_i:\mathbb {R}^{l+r}\rightarrow \mathbb {R}$, for $i=1,\ldots ,m$, such that for all $\zeta \in \mathcal {Z}\subset \mathbb {R}^{l}$, $x\in \mathcal {X}\subset \mathbb {R}^r$, and $y\in \mathcal {Y}(x)\subset \mathbb {R}^n$,

$$\begin{aligned} f(\zeta , x,y)=&\tilde{f}(x,y)+\bar{f}(\zeta , x),&\\ g_i(\zeta , x,y)=&\tilde{g}_i(x,y)+\bar{g}_i(\zeta , x),&i=1,\ldots ,m. \end{aligned}$$

In this paper, we work primarily with constraint-wise uncertainty, which is defined as follows.

#### Definition 4

(*Constraint-wise uncertainty* [4]) For problem (1), the uncertainty is constraint-wise when each uncertain parameter $\zeta$ can be split into blocks $\zeta =\left[ \zeta _0,\ldots , \zeta _{m} \right] \in \mathbb {R}^l$ such that the data of the objective depends only on $\zeta _0\in \mathbb {R}^{l_0}$, the data of the *i*-th constraint depends solely on $\zeta _i\in \mathbb {R}^{l_i}$, and the uncertainty set $\mathcal {Z}=\mathcal {Z}_0 \times \mathcal {Z}_1 \times \ldots \times \mathcal {Z}_{m}$, where $\mathcal {Z}_i\subseteq \mathbb {R}^{l_i}$ is the uncertainty region for $\zeta _i$, for some integers $l_i$, $i=0,\ldots ,m$.

Constraint-wise uncertainty appears in Markov decision process, for example, where it is called rectangularity [18, 21].

Notice that problem (1) does not contain any equality constraint that depends on $\zeta$. The usual way of dealing with such uncertain equalities in (*ARC*) is to eliminate adjustable variables [14, Section 7]. This means that we are implicitly forcing the adjustable variables that are eliminated to obey specific decision rules. This is not allowed in (*RC*). We illustrate this in Example 4 of Supplementary Material Section D.

We will now outline the assumptions used in this paper to express conditions under which $Opt(RC)=Opt(ARC)$.

#### Assumption 1

All the assumptions are with respect to problem (1). Throughout this paper, we assume that

i. There is no equality constraint in problem (1) that depends on $\zeta$.
ii. The uncertainty set $\mathcal {Z}$ is compact.
iii. The uncertainty set $\mathcal {Z}\subset \mathbb {R}^l$ is convex.
iv. $\mathcal {Y}(x)$ is a convex set for each $x\in \mathcal {X}$.
v. $\mathcal {Y}(x)$ is a compact set for each $x\in \mathcal {X}$.
vi. $f(.,x,y)$ and $g_i(.,x,y)$ are concave for each $x\in \mathcal {X}$, $y\in \mathcal {Y}(x)$, and $i=1,\ldots ,m$.
vii. $f(\zeta _0,x,.)$ and $g_i(\zeta _i,x,.)$ are convex for each $x\in \mathcal {X}$, $\zeta \in \mathcal {Z}=\mathcal {Z}_0\times \ldots \times \mathcal {Z}_{m}$, and $i=1,\ldots ,m$.

Assumptions i, iii, iv, vi, and vii are essentially the framework of static robust convex optimization considered in [5].

### Constraint-wise uncertainty

In this subsection, we study problems with constraint-wise uncertainty and provide a set of conditions, under which *Opt*(*RC*) and *Opt*(*ARC*) are equal.

#### Theorem 1

If problem (1) has constraint-wise uncertainty and Assumptions i–vii hold, then $Opt(RC)=Opt(ARC)$.

#### Proof

The line of reasoning is the same as in [4, Theorem 2.1].

**Case I:** Suppose that (*ARC*) does not have a non-adjustable variable. First, we assume that (*RC*) is feasible. So, it is sufficient to show that whenever $\bar{t}\ge Opt(ARC)$, then $\bar{t}\ge Opt(RC)$ (feasibility of (*RC*) implies $Opt(ARC)< \infty$). According to the definitions, we have: 3 and, 4 If $\mathcal {Y} =\emptyset$, it is clear that $Opt(ARC)=Opt(RC)=+\infty$. Now, assume that $\mathcal {Y}\ne \emptyset$. By contradiction, suppose that there is a scalar $\bar{t}$ such that $\bar{t}\ge Opt(ARC)$ and $\bar{t}< Opt(RC)$. Because of the constraint-wise uncertainty, by setting $\beta =(1,0,0,\ldots ,0)^T$, $G_0(\zeta _0,y) =f(\zeta _0,y)$, and $G_i(\zeta _i,y) =g_i(\zeta _i,y)$, for $i=1,\ldots ,m$, and by (4), it follows that
  $$\begin{aligned} \forall y\in \mathcal {Y} \ \ \exists \zeta ^y\in \mathcal {Z} \ \exists i_y\in \{0,\ldots ,m\} : G_{i_y} (\zeta ^y_{i_y},y)-\beta _{i_y} \bar{t}>0. \end{aligned}$$
  Also, continuity implies
  5 $$\begin{aligned} \forall y\in \mathcal {Y} \ \ \exists \epsilon ^y>0\ \exists U_y\ \ \forall z\in U_y: G_{i_y} (\zeta ^y_{i_y},z)-\beta _{i_y}\bar{t}> \epsilon ^y, \end{aligned}$$
  where $U_y$ is the intersection of a 2-norm open ball with a strictly positive radius centered at $y$ with $\mathcal {Y}$. Since $\mathcal {Y}$ is compact, there are $y^k \in \mathcal {Y}$, $k=1,\ldots ,N,$ such that $\mathcal {Y}= \cup _{k=1}^N U_{y^k}$. So,
  6 $$\begin{aligned} \forall k=1,\ldots ,N,\ \forall z\in \mathcal {Y}\ \ \ \max _k G_{i_{y^k}}(\zeta ^{y^k}_{i_{y^k}},z)-\beta _{{i_{y^k}}}\bar{t}>\epsilon , \end{aligned}$$
  where $\epsilon =\min _k \epsilon ^{y^k}$. As a simplification, we set $\zeta ^k=\zeta ^{y^k} _{i_{y^k}}$, $i_k=i_{y^k}$ and
  $$\begin{aligned} f_k(z)=G_{i_k}(\zeta ^k ,z)-\beta _{i_k}\bar{t} \ \ \ \ \ \forall z\in \mathcal {Y}. \end{aligned}$$
  Since $\mathcal {Y}$ is convex and all $f_k(z)$ are convex and continuous on $\mathcal {Y}$ due to Assumption vii, and because $\max _k f_k(z)\ge \epsilon$ for each $z \in \mathcal {Y}$, there are nonnegative weights $\lambda _k$ with $\sum _{k}\lambda _k=1$ such that
  7 $$\begin{aligned} f(z):=\sum _{k}\lambda _k f_k(z)\ge \epsilon \ \ \ \ \ \forall z \in \mathcal {Y}. \end{aligned}$$
  We define
  8 $$\begin{aligned}&w_i=\sum _{k:i_{k}=i}\lambda _k \ \ \ \ i=0,\ldots ,m \nonumber \\&\bar{\zeta }_i=\left\{ \begin{matrix} \sum _{k:i_{k}=i} \frac{\lambda _k}{w_i}\zeta ^{k}, &{} w_i\ne 0 \\ \text{ an } \text{ arbitrary } \text{ point } \text{ in } \ \mathcal {Z}_i, &{} w_i=0 \end{matrix}\right. \nonumber \\&\bar{\zeta }=\left[ \bar{\zeta }_ 0,\ldots ,\bar{\zeta }_ {m} \right] . \end{aligned}$$
  It is clear by convexity of $\mathcal {Z}$ that $\bar{\zeta } \in \mathcal {Z}$. Additionally, due to $\bar{t}\ge Opt(ARC)$, we have 9 which means
  10 $$\begin{aligned} \exists \bar{y}\in \mathcal {Y}: G_i(\bar{\zeta }_i,\bar{y})-\beta _i\bar{t}\le 0,\ \ \ \ \ i=0,\ldots ,m. \end{aligned}$$
  Also, we know that for each $i=0,\ldots ,m$, the functions $G_i(\zeta _i,\bar{y})$ are concave in $\zeta _i$ due to Assumption vi. Hence, for all $i=0,\ldots ,m$, and $w_i> 0$
  $$\begin{aligned} G_i(\bar{\zeta }_i,\bar{y})-\beta _i\bar{t}&=G_i \left( \sum _{k:i_{k}=i} \frac{\lambda _k}{w_i}\zeta ^k,\bar{y}\right) -\beta _i\bar{t} \nonumber \\&\ge \sum _{k:i_k=i}\frac{\lambda _k}{w_i}G_i( \zeta ^k,\bar{y}) -\beta _i\bar{t} \nonumber = \sum _{k:i_k=i}\frac{\lambda _k}{w_i}f_k(\bar{y}). \nonumber \end{aligned}$$
  Summing over the indices results in
  11 $$\begin{aligned} \sum _{\begin{array}{c} i=1 \\ w_i \ne 0 \end{array}}^m w_i \left( G_i(\bar{\zeta }_i,\bar{y})-\beta _i\bar{t} \right) \ge \sum _{k=1}^N\lambda _kf_k(\bar{y}). \end{aligned}$$
  By applying (7) and (10), the above inequality contradicts $\epsilon >0$. Now we consider the case where (*RC*) is not feasible, which means $Opt(RC)=+\infty$. To prove equality of (*RC*) and (*ARC*) with respect to the worst-case objective value, it is sufficient to show that there is no $\bar{t} \in \mathbb {R}$ such that $\bar{t}\ge Opt(ARC)$. So, the same argument used in the previous part implies that $Opt(ARC)=+\infty$.
**Case II**: Now, we consider a general case, where (*ARC*) contains the non-adjustable variable $x$. As proved in Case I, for any $x\in \mathcal {X}$,
  12 $$\begin{aligned} \begin{matrix} \sup _{\zeta \in \mathcal {Z}}&{} \inf _{y(\zeta ) \in \mathcal {Y}(x)}&{} f(\zeta ,x,y(\zeta )) \ &{} \\ &{} \text{ s.t. } &{} g_i(\zeta ,x,y(\zeta ))\le 0, &{}\ \ i=1,\ldots ,m, \end{matrix} \end{aligned}$$
  and
  13 $$\begin{aligned} \begin{matrix} \inf _{y\in \mathcal {Y}(x)} &{} \sup _{\zeta \in \mathcal {Z}}\ f(\zeta _0 ,x,y)\ &{} &{}\\ \text{ s.t. } &{} g_i(\zeta _i ,x,y(\zeta _0,\ldots ,\zeta _m))\le 0, &{} \forall \zeta _i \in \mathcal {Z}_i, &{} i=1,\ldots ,m, \end{matrix} \end{aligned}$$
  have the same optimal value. Therefore, taking the infimum over all $x\in \mathcal {X}$ results in $Opt(RC)=Opt(ARC)$. $\square$

Theorem 1 extends the results for linear problems, [6, Proposition 2.1], to nonlinear ones. In Supplementary Material Section B, we replace Assumption v in Theorem 1 with two other assumptions in order to provide another set of conditions under which $Opt(RC)=Opt(ARC)$.

#### Remark 1

For a problem with fixed recourse and constraint-wise uncertainty, Assumption ii implies equality of the objective values of (*RC*) and (*ARC*). The complete proof can be found in Supplementary Material Section A. Even though in this case the resulting (*RC*) is *NP*-hard in general, there are cases for which (*RC*) is tractable, for instance see [3, Section 1.4].

### Non-constraint-wise uncertainty

Section 2.2 focuses on constraint-wise uncertainty. The question is what can be concluded for a problem in which some, but not all, of the uncertain parameters are constraint-wise. Consider the following problem:where $\zeta =(\zeta _0,\ldots ,\zeta _{m})\in \mathcal {Z}= \mathcal {Z}_0\times \ldots \times \mathcal {Z}_{m}$ and $\alpha \in \mathcal {A}\subseteq \mathbb {R}^d$ are uncertain parameters ($\zeta$ is constraint-wise and $\alpha$ is non-constraint-wise). This problem has a hybrid uncertainty, so we cannot use the results in Section 2.2 to deduce equality of the optimal values of the hybrid robust counterpart (*HRC*) and the corresponding hybrid adjustable robust counterpart (*HARC*). However, the following corollary states that if in such a case the same set of conditions as in Theorem 1 hold with respect to the constraint-wise uncertain parameters, then there exists an optimal decision rule that is a function of only the non-constraint-wise uncertain parameters. In other words, the two problemsandhave the same optimal objective values. We denote the optimal objective values of (*HARC*) and $(HARC_{\alpha })$ by *Opt*(*HARC*) and $Opt(HARC_{\alpha })$, respectively.

#### Corollary 1

Suppose that for all $\alpha \in \mathcal {A}$, the assumptions of Theorem 1 hold with respect to $\zeta , \ x,\ y$. Then, $Opt(HARC)=Opt(HARC_{\alpha })$.

#### Proof

By fixing $\alpha \in \mathcal {A}$ and $x\in \mathcal {X}$ and applying Theorem 1, the optimal objective value ofandare equal. The result follows from taking the supremum over $\alpha \in \mathcal {A}$ and infimum over $x\in \mathcal {X}$. $\square$

Corollary 1 can be used to reduce the complexity of solving adjustable robust optimization problems. This is because in order to solve (*HARC*), one needs to find an optimal decision rule with respect to both $\alpha$ and $\zeta$, but, we can ensure the existence of an optimal decision rule that only depends on $\alpha$ by applying this corollary.

It is important to note that if we restrict ourselves to one class of decision rules, e.g., affine decision rules, as is customary, then Corollary 1 does not necessarily guarantee the existence of an optimal affine decision rule that only depends on $\alpha$. The following corollary, however, states that if the problem has fixed recourse with respect to the constraint-wise uncertain parameter $\zeta$ and we use a specific class of decision rules that are separable with respect to $\zeta$ and $\alpha$, then there exists an optimal decision rule that depends only on $\alpha$.

Let us denote by $\bar{y}_\omega (\alpha ){:\mathbb {R}^d\rightarrow \mathbb {R}}$ a function of $\alpha$ that belongs to a specific class parametrized by $\omega$. One of the examples for $\bar{y}_\omega \left( \alpha \right)$ is a polynomial. In this case, $\omega$ could be the vector of coefficients for the monomials.

#### Corollary 2

Assume that in (*HARC*),

14 $$\begin{aligned} g_i(\zeta _i,\alpha ,x,y)= \tilde{g}_i(\zeta _i,x)+\bar{g}_i(\alpha ,x,y), i=0,\ldots ,m, \end{aligned}$$

where $g_0(\zeta _0,\alpha ,x,y)=f(\zeta _0,\alpha ,x,y)$ and $\tilde{g}_i(\zeta _i,x)$ and $\bar{g}_i(\alpha ,x,y)$ are continuous for $i=0,\ldots ,m$. Also, assume that we restrict the decision rules to be in the form of $y\left( \zeta \right) +\bar{y}_\omega \left( \alpha \right) ,$ where $y(.):\mathbb {R}^l\longrightarrow \mathbb {R}^n$. Then the optimal objective value of (*HRC*) when using this decision rule is equal to that of using decision rule $y+\bar{y}_\omega \left( \alpha \right)$.

#### Proof

Consider the following problem:15By defining $\bar{\mathcal {Y}}\left( x,\omega \right) =\cap _{\alpha \in \mathcal {A}} \left[ \mathcal {Y}\left( x\right) -\bar{y}_\omega \left( \alpha \right) \right]$ and

$$\begin{aligned} \hat{g}_i\left( x,\omega , y\left( \zeta \right) \right) =\sup _{\alpha \in \mathcal {A}}\bar{g}_i\left( \alpha ,x,y\left( \zeta \right) +\bar{y}_\omega \left( \alpha \right) \right) , i=0,\ldots ,m, \end{aligned}$$

accordingly, we obtain an optimal objective value for (15) that is equal to the optimal objective value of16which is the adjustable robust counterpart related to the following robust problem:17In accordance with Remark 1, (16) and (17) have the same optimal objective value. Using the definitions of $\bar{\mathcal {Y}}\left( x,\omega \right)$ and $\hat{g}_i(x,\omega , y)$, $i=0,\ldots ,m$, we can easily see that the optimal objective value of (17) is equal to the optimal objective value of18 So, we have proved that the optimal objective value of (15) and (18) are the same. This means that the use of $y\left( \zeta \right) +\bar{y}_\omega \left( \alpha \right)$ and $y+\bar{y}_\omega \left( \alpha \right)$ as decision rules yields the same approximation of the optimal objective values. $\square$

In Corollary 2, $y(\zeta )$ is a general function. For instance, if we assume that $\bar{y}_\omega \left( \alpha \right)$ lies in the class of affine functions, even for a general $y(\zeta )$, the optimal objective value is independent from $\zeta$. The other example is when both $y(\zeta )$ and $\bar{y}_\omega \left( \alpha \right)$ are affine, which means that the decision rule is affine. We consider this case in the next corollary.

#### Corollary 3

Suppose that in (*HARC*) the constraints and objective functions satisfy (14). Then, using an affine decision rule, $y( \alpha )=u +W\alpha$ or $y(\zeta , \alpha )=u+V\zeta +W\alpha$, where $u \in \mathbb {R}^n$, $V\in \mathbb {R}^{n \times l}$, and $W\in \mathbb {R}^{n\times d}$, yields the same approximate optimal value.

Corollary 3 mentions two different problems for approximating (*HARC*): one considers $y( \alpha )=u +W\alpha$ as the form of decision rule and the other $y(\zeta , \alpha )=u+V\zeta +W\alpha$. We denote the optimal objective values of the former affinely adjustable robust counterparts by $Opt(AARC_{ \alpha })$ and $Opt(AARC_{\zeta , \alpha })$, respectively. Then, in general, for problem (*HRC*), we have

19 $$\begin{aligned} Opt(HARC)\le Opt(AARC_{\zeta , \alpha }) \le Opt(AARC_{ \alpha }) \le Opt(HRC). \end{aligned}$$

In this section, we discussed conditions that turn inequalities in (19) into equalities. Theorem 1 provides a set of conditions under which all of the inequalities can be replaced by equalities. In addition, under a similar set of conditions as in that theorem, Corollary 3 ensures us that the middle inequality in (19) turns into an equality. Other sets of conditions for which $Opt(HARC)=Opt(AARC_{\zeta , \alpha })$ are proposed in [8, 11, 16]. In Supplementary Material Section D, we provide some examples to show that these inequalities can be strict.

## Application

One application of the results derived in Sect. 2 is for the following problem:

$$\begin{aligned} \begin{aligned} \inf _{x\in \mathcal {X}}\; \inf _{y\in \mathcal {Y}(x)}&\ f(x,y)\\ \text{ s.t. }&g_j(\alpha ,x,y)\le 0,\;\forall \alpha \in \mathcal {A},\;j=1,\ldots ,m,\\&h_i(\zeta _i,x, y)\le 0,\;\forall \zeta _i\in \mathcal {Z}_i,\; i=1,\ldots ,I,\\&p_k(\theta _k,x, y)\le 0,\;\theta _k\in \mathcal {T}_k,\; k=1,\ldots ,K, \end{aligned} \end{aligned}$$

where $g_j(\alpha ,x,y)$, $j=1,\ldots ,m,$ is a continuous function, the convex quadratic function $h_i$ is defined as

$$\begin{aligned} h_i(\zeta _i,x, y)=\left( \begin{array}{c} x\\ y \end{array}\right) ^TA_i(\zeta _i)\left( \begin{array}{c} x\\ y \end{array}\right) +b_i(\zeta _i)^T\left( \begin{array}{c} x\\ y \end{array}\right) +c_i(\zeta _i), \end{aligned}$$

and the conic quadratic function $p_k$ is defined as

$$\begin{aligned} p_k(\theta _k,x, y)=\sqrt{\left( \begin{array}{c} x\\ y \end{array}\right) ^TB_k(\theta _k)\left( \begin{array}{c} x\\ y \end{array}\right) } +d_k(\theta _{k})^T\left( \begin{array}{c} x\\ y \end{array}\right) +e_k(\theta _{k}), \end{aligned}$$

where $\alpha \in \mathcal {R}^{l}$, $\zeta _i\in \mathcal {R}^{l_i}$, and $\theta _k\in \mathcal {R}^{l_{I+k}}$ are the uncertain parameters for some integers $l, l_{i}, l_{I+k}$, $i=1,\ldots ,I$, $k=1,\ldots ,K,$ and $x$ and $y$ are non-adjustable and adjustable variables, respectively. We assume that the matrices $A_i(\zeta _i)$ and $B_k(\theta _k)$ are positive semi-definite for all $\zeta _i \in \mathcal {Z}_i$ and $\theta _k\in \mathcal {T}_k$, $i=1,\ldots ,I$, $k=1,\ldots ,K$. Also, we assume that $A_i(\zeta _i),\ b_i(\zeta _i),\ c_i(\zeta _i)$, $B_k(\theta _k),\ d_k(\theta _{k})$, and $e_k(\theta _{k})$ are affine in $\zeta _i$ and $\theta _k$, $i=1,\ldots ,I$, $k=1,\ldots ,K$, respectively.

This type of problem arises, for example, when a part of the problem is related to multi-stage mean-variance portfolio optimization [15], in which the asset return mean and covariance matrix are uncertain and these uncertainties only occur in the objective function (hence the problem has constraint-wise uncertainty).

If the uncertainty over $\alpha$ is constraint-wise and $g_j(\alpha ,x,y)$ is concave in $\alpha$ and convex in $y$, $j=1,\ldots ,m$; $\mathcal {A}$, $\mathcal {Z}_i$ and $\mathcal {T}_k$ are convex, $i=1,\ldots ,I$, $k=1,\ldots ,K$; and $\mathcal {Y}(x)$ is compact and convex for all $x\in \mathcal {X}$, then by Theorem 1, the optimal values of the corresponding static and adjustable robust problems are equal, because $h_i$ and $p_k$ are convex in $y$ and concave in $\zeta _i$ and $\theta _k$, $i=1,\ldots ,I$, $k=1,\ldots ,K$, respectively. Moreover, if the uncertainty over $\alpha$ is not constraint-wise, then by Corollary 1, an optimal *y* exists for the corresponding adjustable robust counterpart that is independent of $\zeta _i$ and $\theta _k$, $i=1,\ldots ,I$, $k=1,\ldots ,K$.

## Conclusion

In this paper, we show that for a class of constraint-wise uncertain convex optimization problems, the static robust optimal solution is also optimal for adjustable robust problems. This class consists of problems that are convex with respect to the adjustable variables and concave with respect to the uncertain parameters, and that have a convex compact uncertainty set and adjustable variables that lie in a convex compact set.

This result does not hold for problems where just some of the uncertain parameters are constraint-wise. We prove that under a set of assumptions similar to the pure constraint-wise case, there exists an optimal decision rule that does not depend on the constraint-wise uncertain parameters. Also, we show that for one class of problems, restricting decision rules to be affine and independent of the constraint-wise uncertain parameters yields the same optimal objective value as in cases where the decision rules are affine and dependent on both the constraint-wise and non-constraint-wise uncertain parameters.

Lastly, we prove that for adjustable robust optimization problems with convex quadratic and/or conic quadratic constraints, if the uncertainty in the quadratic constraints is constraint-wise, then optimal adjustable variables exist that are independent of the constraint-wise uncertain parameters.

## Electronic supplementary material

Below is the link to the electronic supplementary material.

Supplementary material 1 (pdf 231 KB)
