# Supplementary material for: When are static and adjustable robust optimization problems with constraint-wise uncertainty equivalent?
Source: Math Program. 2017 Jun 12;170(2):555–68. doi: 10.1007/s10107-017-1166-z (PMC6435025; doi:10.1007/s10107-017-1166-z)
Supplement: Supplementary file 1 — Supplementary material 1 (pdf 231 KB) [file 10107_2017_1166_MOESM1_ESM.pdf]

Math. Program., Ser. A,
DOI 10.1007/s10107-017-1166-z

Electronic Supplementary Material to the paper "When are static and adjustable robust optimization problems equivalent with constraint-wise uncertainty?"

Ahmadreza Marandi · Dick den Hertog

This supplementary material is divided into four parts. In the first part, we prove Remark 1. In the second part, we provide a set of conditions, different than the set provided in Section 2.2, under which $Opt(ARC) = Opt(RC)$. In the third part, we provide some examples that illustrate the results from Section 2. The fourth part presents several examples to show that the assumptions in Theorem 1 are essential.

A. Proof of Remark 1

Assume that in problem (*1*), the uncertainty is constraint-wise and the uncertainty set is compact. If (ARC) has fixed recourse, then $Opt(RC) = Opt(ARC)$.

Proof First, we suppose problem (1) does not contain any non-adjustable variables. According to the definitions of (RC) and (ARC), we have $Opt(ARC) \leq Opt(RC)$. That means that if (RC) is unbounded, then $Opt(RC) = Opt(ARC) = -\infty$. Now, if (RC) is not unbounded, we show that $Opt(ARC) \geq Opt(RC)$.

Since (ARC) has fixed recourse, we can simplify (RC) to the following problem:

$$\begin{aligned} \inf_{y\in\mathcal{Y},\ t} \quad & t \\ \text{s.t.} \quad & \tilde{f}(y) + \sup_{\zeta_0\in\mathcal{Z}_0} \bar{f}(\zeta_0) \leq t \\ & \tilde{g}_i(y) + \sup_{\zeta_i\in\mathcal{Z}_i} \bar{g}_i(\zeta_i) \leq 0, \quad i = 1, ..., m. \end{aligned} \tag{20}$$

Since $\bar{f}$ and $\bar{g}_i$, $i = 1, ..., m$, are continuous, and the uncertainty is constraint-wise, and $\mathcal{Z}$ is non-empty and compact, there is a point $\bar{\zeta} = [\bar{\zeta}_0, ..., \bar{\zeta}_m] \in \mathcal{Z}$ where $\bar{\zeta}_0$ is an optimal solution of $\sup_{\zeta_0\in\mathcal{Z}_0} \bar{f}(\zeta_0)$, and $\bar{\zeta}_i$ is an optimal solution of $\sup_{\zeta_i\in\mathcal{Z}_i} \bar{g}_i(\zeta_i)$, for all $i = 1, ..., m$. According to the definition of (ARC), $Opt(ARC) \geq q$, where

$$\begin{aligned} q := \inf_{\substack{y(\bar{\zeta}) \in \mathcal{Y} \\ t(\bar{\zeta})}} \quad & t(\bar{\zeta}) \\ & \tilde{f}(y(\bar{\zeta})) + \bar{f}(\bar{\zeta}_0) \leq t(\bar{\zeta}) \\ & \tilde{g}_i(y(\bar{\zeta})) + \bar{g}_i(\bar{\zeta}_i) \leq 0, \quad i = 1, ..., m, \end{aligned} \tag{21}$$

A. Marandi
Tilburg School of Economics and Management
Tilburg University, The Netherlands
E-mail: a.marandi@uvt.nl

D. den Hertog
Tilburg School of Economics and Management
Tilburg University, The Netherlands
E-mail: D.denHertog@uvt.nl

which is equivalent to (20). This implies that if (RC) is infeasible, so is (21), and therefore $Opt(ARC) = Opt(RC) = +\infty$. On the other hand, if (RC) is feasible, then $Opt(RC) = q \leq Opt(ARC)$. So, the equality of the optimal objective values of (ARC) and (RC) has been proved.

Now, for the general case in which (ARC) contains a non-adjustable variable x, we have to solve:

$$\begin{array}{l} \inf_{x\in\mathcal{X}} \sup_{\zeta\in\mathcal{Z}} \inf_{y(\zeta)\in\mathcal{Y}(x)} f(\zeta,x,y(\zeta)) \\ \qquad\qquad\qquad g_i(\zeta,x,y(\zeta)) \leq 0, \quad i=1,...,m. \end{array} \tag{22}$$

According to the first part of the proof, we have that for each $x \in \mathcal{X}$, the objective value of

$$\begin{array}{ll} \sup_{\zeta\in\mathcal{Z}} \inf_{y(\zeta)\in\mathcal{Y}(x)} & f(\zeta,x,y(\zeta)) \\ \qquad\qquad \text{s.t.} & g_i(\zeta,x,y(\zeta)) \leq 0, \quad i=1,...,m \end{array} \tag{23}$$

is equal to the objective value of

$$\begin{array}{ll} \inf_{y\in\mathcal{Y}(x)} \sup_{\zeta\in\mathcal{Z}} & f(\zeta_0,x,y) \\ \qquad \text{s.t.} & g_i(\zeta_i,x,y) \leq 0, \quad \forall\zeta_i \in \mathcal{Z}_i, \quad i=1,...,m. \end{array} \tag{24}$$

It follows, then, that the optimal objective value of problem (22) equals that of

$$\begin{array}{ll} \inf_{x\in\mathcal{X},y\in\mathcal{Y}(x)} \sup_{\zeta\in\mathcal{Z}} & f(\zeta_0,x,y) \\ \qquad \text{s.t.} & g_i(\zeta_i,x,y) \leq 0, \quad \forall\zeta_i \in \mathcal{Z}_i, \; i=1,...,m. \end{array} \tag{25}$$

Therefore, $Opt(ARC) = Opt(RC)$. □

B. Another set of conditions under which $Opt(ARC) = Opt(RC)$

In this section, we prove that $Opt(ARC) = Opt(RC)$ under a set of conditions different than those provided in Theorem 1. For this theorem, without loss of generality, we assume that (RC) is

$$\begin{array}{ll} \inf_{x\in\mathcal{X}} \inf_{y\in\mathcal{Y}(x)} & c^T y \\ \qquad \text{s.t.} & g_i(\zeta_i,x,y) \leq 0, \quad i=0,...,m, \quad \forall\zeta_i \in \mathcal{Z}_i, \end{array} \tag{26}$$

where $c \in \mathbb{R}^r$ is certain, and for $i = 0,...,m$,

$$\mathcal{Z}_i = \{\zeta_i : \; h_{ik}(\zeta_i) \leq 0, \; k = 1,...,K_i\}.$$

In what follows, the relative interior of a set S and domain of a function $f(.)$ are denoted by relint(S) and dom$(f(.))$, respectively.

Theorem. *Assume that for problem (26) the following assumptions hold:*

(a) $h_{ik}(.)$ is convex, $i = 0,...,m$, $k = 1,...,K_i$,
(b) There exists $(\zeta_0,...,\zeta_m)$ such that $h_{ik}(\zeta_i) < 0$ for all $i = 0,...,m$, $k = 1,...,K_i$;
(c) For each $x \in \mathcal{X}$ and $\zeta \in \mathcal{Z}$;

$$\bigcap_{i=1}^{m} relint(dom(g_i(\zeta_i,x,.))) \bigcap relint(\mathcal{Y}(x)) \neq \emptyset.$$

Additionally, if Assumptions iv, vi, and vii hold, then $Opt(ARC) = Opt(RC)$.

Proof Consider the (ARC) corresponding to (26) to be

$$\begin{array}{l} \inf_{x\in\mathcal{X}} \sup_{[\zeta_0,...,\zeta_m]\in\mathcal{Z}} \inf_{y(\zeta)\in\mathcal{Y}(x)} c^T y(\zeta) \\ \qquad\qquad\qquad \text{s.t. } g_i(\zeta_i,x,y(\zeta)) \leq 0, \quad i=0,...,m. \end{array} \tag{27}$$

By [2, Lemma 9] (because of Assumptions iv and vii and assumption (c)), the optimal value of (27) is equal to

$$\begin{aligned}
\inf_{x\in\mathcal{X}} \sup_{\substack{u\,\in\,\mathbb{R}^{m+1}_{\leq}\\ \{v^i\},\,v^{m+1}}} \sup_{\zeta=[\zeta_0,\dots,\zeta_m]} \quad & \sum_{i=0}^{m} u_i g_i^*\left(\zeta_i, x, \frac{v^i}{u_i}\right) + u_{m+1}\delta^*_{\mathcal{Y}(x)}\left(\frac{v^{m+1}}{u_{m+1}}\right) \\
\text{s.t.} \quad & \sum_{i=0}^{m+1} v^i = c, \\
& h_{ik}(\zeta_i) \leq 0, \quad i=0,...,m,\ k=1,...,K_i,
\end{aligned} \tag{28}$$

where $\delta^*_{\mathcal{Y}(x)}\left(\frac{v^{m+1}}{u_{m+1}}\right) = \sup_{y\in\mathcal{Y}(x)} \frac{y^T v^{m+1}}{u_{m+1}}$ and

$$g_i^*\left(\zeta_i, x, \frac{v^i}{u_i}\right) = \sup_{y\in dom(g_i(\zeta_i,x,.))} \left\{\frac{y^T v^i}{u_i} - g_i\left(\zeta_i, x, y\right)\right\}.$$

Problem (28) has the same optimal objective value as

$$\begin{aligned}
\inf_{x\in\mathcal{X}} \sup_{\substack{u\,\in\,\mathbb{R}^{m+1}_{\leq}\\ \{v^i\},\,v^{m+1}}} \sup_{w^i} \quad & \sum_{i=0}^{m} u_i g_i^*\left(\frac{w^i}{u_i}, x, \frac{v^i}{u_i}\right) + u_{m+1}\delta^*_{\mathcal{Y}(x)}\left(\frac{v^{m+1}}{u_{m+1}}\right) \\
\text{s.t.} \quad & \sum_{i=0}^{m+1} v^i = c, \\
& -u_i h_{ik}\left(\frac{w_i}{u_i}\right) \leq 0, \quad i=0,...,m,\ k=1,...,K_i,
\end{aligned}$$

which is the dual of (26), with the same optimal objective values according to [13, Theorem 1] (because the uncertainty is constraint-wise and assumptions (a) and (b), as well as Assumptions vi and vii, hold). So, $Opt(ARC) = Opt(RC)$. □

C. Illustrative examples

Example 1 **(Illustrating Theorem 1)** Consider the following problem:

$$\begin{aligned}
\min \quad & y_1 + y_2 \\
\text{s.t.} \quad & \ln(\zeta) y_1^2 + y_2^2 \leq 3, \\
& y_1^2 + y_2^2 \leq 4,
\end{aligned}$$

where $\zeta \in \mathcal{Z} = [1,4]$ is an uncertain parameter and $y = (y_1, y_2)$ is an adjustable variable.

For this example, $Opt(RC) = Opt(ARC)$ because by defining

$$\mathcal{Y} = \left\{y \;\middle|\; y_1^2 + y_2^2 \leq 4\right\},$$

we create the conditions under which the assumptions of Theorem 1 will hold for this problem. Since $\ln(\zeta)$ is an increasing function, (RC) is as follows:

$$\begin{aligned}
\min \quad & y_1 + y_2 \\
\text{s.t.} \quad & \ln(4) y_1^2 + y_2^2 \leq 3, \\
& y_1^2 + y_2^2 \leq 4,
\end{aligned}$$

which has an optimal value of $-\sqrt{\frac{3\ln(4)}{1+\ln(4)}}\left(\frac{1}{\ln(4)} + 1\right)$.

Even though $Opt(RC) = Opt(ARC)$, by using the symmetry bound introduced in [9], which is $(1+\rho)\, Opt(RC) \leq Opt(ARC) \leq Opt(RC)$, where

$$\rho = \min\left\{\alpha \geq 0 \;\middle|\; \mathcal{Z} - (1-\alpha)\frac{5}{2} \subset \mathbb{R}_+\right\} = \frac{3}{5},$$

one gets $\left(\frac{8}{5}\right) Opt(RC) \leq Opt(ARC) \leq Opt(RC)$.

This example shows that the symmetry bound is not tight in the presence of constraint-wise uncertainty, even when the problem only has one uncertain parameter. □

Example 2 **(Illustrating Remark 1)** Consider the uncertain problem

$$\begin{aligned} \min\ & y^2 + x^3 \\ \text{s.t.}\ & y^3 + \zeta^3 x \le 0, \\ & y^2 + x^2 \le 8, \\ & |x| \le 1, \end{aligned}$$

where $\zeta \in \mathcal{Z} = [-2, 2]$ is an uncertain parameter, y is an adjustable variable, and x is a non-adjustable variable. For this problem,

$$\mathcal{X} = [-1, 1], \ \ \mathcal{Y}(x) = \left\{ y \ \middle|\ y^2 + x^2 \le 8 \right\}, \ \ \forall x \in \mathcal{X}.$$

First, we use Remark 1 to calculate $Opt(ARC)$, because the relevant assumptions hold for this problem. According to this remark, $Opt(ARC) = Opt(RC)$. Since ζ^3 is an increasing function, (RC) is equivalent to

$$\begin{aligned} \min\ & y^2 + x^3 \\ \text{s.t.}\ & y^3 + 8x \le 0, \\ & y^3 - 8x \le 0, \\ & y^2 + x^2 \le 8, \\ & |x| \le 1. \end{aligned}$$

It is easy to verify that $Opt(RC) = 0$. Now we solve the (ARC) problem

$$\begin{aligned} \min_{x\in\mathcal{X}} \ \max_{\zeta\in\mathcal{Z}} \ \min_{y(\zeta)} \ & y(\zeta)^2 + x^3 \\ \text{s.t.}\ & y(\zeta)^3 + \zeta^3 x \le 0, \\ & y(\zeta)^2 + x^2 \le 8 \end{aligned}$$

directly. First, we solve

$$\begin{aligned} z^*(\zeta, x) := \ \min_{y(\zeta)} \ & y(\zeta)^2 \\ \text{s.t.}\ & y(\zeta)^3 + \zeta^3 x \le 0, \\ & y(\zeta)^2 + x^2 \le 8 \end{aligned}$$

for each $\zeta \in \mathcal{Z}$ and $x \in \mathcal{X}$. It is clear that

$$z^*(\zeta, x) = \begin{cases} \left(\sqrt[3]{-\zeta^3 x}\right)^2, & \zeta x \ge 0, \\ 0, & \text{otherwise.} \end{cases}$$

Hence, $Opt(ARC) = \min_{x\in\mathcal{X}} x^3 + \max_{\zeta\in\mathcal{Z}} z^*(\zeta, x)$. Therefore, we need the optimal objective value of $\max_{\zeta\in[-2,2]} \ z^*(\zeta, x)$ for each $x \in \mathcal{X}$. By checking two cases $x \ge 0$ and $x < 0$, we find $4\sqrt[3]{x^2}$ as its optimal objective value. Hence, $Opt(ARC) = min_{x\in[-1,1]} x^3 + 4\sqrt[3]{x^2} = 0$. □

Hitherto, we have studied examples regarding constraint-wise uncertainty. Now, we consider an example possessing hybrid uncertainty.

Example 3 **(Hybrid uncertainty)** Consider the following uncertain problem:

$$\begin{aligned} \min_{y,x} & - x \\ \text{s.t.}\ & (1 - 2\alpha)x + y \ge \zeta, \\ & \alpha x - y \ge 0, \\ & x \le 1, \end{aligned} \tag{29}$$

where $\alpha \in [0, 1]$ is a non-constraint-wise and $\zeta \in [-1, 0]$ a constraint-wise uncertain parameter, y is an adjustable variable, and x is a non-adjustable variable.

Corollary 1 shows that there exists an optimal decision rule for $(HARC)$ that is independent of ζ. In this example, we check the inequalities in (19). First, we find the optimal objective values

of the static and adjustable robust counterparts corresponding to (29). After that, we discuss the dependency of the optimal decision rules on the uncertain parameters in the adjustable robust optimization problem.

To calculate the optimal value of the robust counterpart corresponding to (29), it is sufficient to solve the following problem:

$$\begin{aligned} q^*_{RC} = \min_{y,x} \; & -x \\ \text{s.t.} \; & x + y \geq 0, \\ & -x + y \geq 0, \\ & -y \geq 0, \\ & x - y \geq 0, \\ & x \leq 1, \end{aligned}$$

because the constraints in (29) are linear with respect to the uncertain parameters α and ζ. This means that $(0, 0)$ is the only robust feasible solution of (29). Hence, $q^*_{RC} = 0$.

The adjustable robust counterpart corresponding to (29) is as follows:

$$\begin{aligned} q^*_{ARC} = \min_{x} \; \max_{(\alpha,\zeta)\in\mathcal{Z}} \; \min_{y(\alpha,\zeta)} \; & -x \\ \text{s.t.} \; & (1-2\alpha)x + y(\alpha,\zeta) \geq \zeta, \\ & \alpha x - y(\alpha,\zeta) \geq 0, \\ & x \leq 1, \end{aligned} \tag{30}$$

where (α, ζ) is the uncertain parameter and $\mathcal{Z} = [0, 1] \times [-1, 0]$ is the uncertainty set. According to the last constraint, $q^*_{ARC} \geq -1$. Fixing $x = 1$, we have

$$\zeta + 2\alpha - 1 \leq y(\alpha, \zeta) \leq \alpha, \tag{31}$$

which means that $q^*_{ARC} = -1$ by choosing $y^*(\alpha, \zeta) = \zeta + 2\alpha - 1$ as the optimal decision rule, which depends on both α and ζ. However, $y^{**}(\alpha, \zeta) = \alpha$ is another optimal decision rule for (30), which is independent of ζ. We thus show by this discussion that for (29) there is only one strict inequality in (19):

$$-1 = Opt(HARC) = Opt(AARC_{\zeta,\alpha}) = Opt(AARC_{\alpha}) < Opt(HRC) = 0. \quad \square$$

D. Counterexamples when one of the conditions is not satisfied

In this section, we consider examples in which all of the assumptions of Theorem 1 are satisfied except one. Each example is associated with the assumption not satisfied.

Example 4 **(Assumption i)** Consider the following problem, in which Assumption i is not satisfied because there is an equality constraint that is dependent on ζ:

$$\begin{aligned} \min \; & -y_1 \\ \text{s.t.} \; & \zeta y_1 + y_2 = 1, \\ & 0 \leq y_1, y_2 \leq 10, \end{aligned} \tag{32}$$

where $\zeta \in [1, 2]$. It is clear that $Opt(RC) = 0$, since $(0, 1)$ is the only robust feasible solution.

To calculate the optimal value of the corresponding (ARC), we eliminate the equality constraint in (32) and reach the following adjustable robust problem:

$$\begin{aligned} \max_{\zeta\in\mathcal{Z}} \; \min_{y_1(\zeta)} \; & -y_1(\zeta) \\ \text{s.t.} \; & 0 \leq y_1(\zeta) \leq \frac{1}{\zeta}. \end{aligned} \tag{33}$$

It is clear that the optimal value of (33) is $\max_{\zeta\in[1,2]} -\frac{1}{\zeta} = -\frac{1}{2}$. Hence, $Opt(ARC) < Opt(RC)$. These optimal values are different because in the elimination, we use the decision rule $y_2 = 1 - \zeta y_1$, which is not allowed in the corresponding (RC). $\square$

Example 5 **(Assumption ii)** Consider the following problem

$$\begin{aligned} \min \quad & -y^2 \\ \text{s.t.} \quad & y \le \zeta, \end{aligned} \tag{34}$$

where $\zeta \le 0$. This problem does not satisfy Assumption ii, since the uncertainty set is not compact. It is clear that $Opt(RC) = +\infty$, because (RC) is infeasible. However, (ARC) is feasible and $Opt(ARC) = -\infty$. □

Example 6 **(Constraint-wise uncertainty)** Ben-Tal et al. [4] consider the following uncertain problem:

$$\begin{aligned} \min \quad & -x \\ s.t. \quad & (1-2\zeta)x + y \ge 0, \\ & \zeta x - y \ge 0, \\ & 0 \le x \le 1, \\ & |y| \le 2, \end{aligned}$$

where $\zeta \in [0,1]$ is an uncertain parameter, y is an adjustable variable, and x is a non-adjustable variable. It is easy to check that all assumptions hold except "constraint-wise uncertainty". The corresponding (RC) can be reformulated as

$$\begin{aligned} \min \quad & -x \\ s.t. \quad & x + y \ge 0, \\ & x - y \ge 0, \\ & -x + y \ge 0, \\ & -y \ge 0, \\ & 0 \le x \le 1, \\ & |y| \le 2. \end{aligned}$$

It can easily be verified here that $Opt(RC) = 0$. The corresponding (ARC) is as follows:

$$\begin{aligned} \min_x \max_\zeta \min_{y(\zeta)} \quad & -x \\ s.t. \quad & (1-2\zeta)x + y(\zeta) \ge 0, \\ & \zeta x - y(\zeta) \ge 0, \\ & 0 \le x \le 1, \\ & |y(\zeta)| \le 2. \end{aligned} \tag{35}$$

Similar to the discussion in Example 3, we can verify that $Opt(ARC) = -1$, which means $Opt(ARC) < Opt(RC)$. □

Example 7 **(Assumption iii)** Consider the problem $\min_{y\in\mathcal{Y}} \zeta y$, where $\zeta \in \mathcal{Z}$ is the uncertain parameter, $\mathcal{Z} = \{-1, 2\}$ is the uncertainty set, and $\mathcal{Y} = [-1, 1]$. It is clear that all the assumptions of Theorem 1 hold except iii. A straightforward calculation leads us to

$$\begin{aligned} Opt(RC) &= \min_{y\in\mathcal{Y}} \max_{\zeta\in\mathcal{Z}} \ \zeta y = \min\{\min_{y\in[0,1]} \max_{\zeta\in\mathcal{Z}} \ \zeta y, \ \min_{y\in[-1,0]} \max_{\zeta\in\mathcal{Z}} \ \zeta y\} \\ &= \min\{\min_{y\in[0,1]} 2y, \ \min_{y\in[-1,0]} -y\} \\ &= 0. \end{aligned}$$

And

$$Opt(ARC) = \max_{\zeta\in\mathcal{Z}} \min_{y(\zeta)\in X} \ \zeta y(\zeta) = \max\{\min_{y\in X} -y, \min_{y\in X} 2y\} = \max\{-1, -2\} = -1.$$

So, $Opt(ARC) < Opt(RC)$. However, if we replace $\mathcal{Z}$ with $Conv(\mathcal{Z})$, then $Opt(RC)$ remains the same but $Opt(ARC)$ becomes zero, which shows that convexity of $\mathcal{Z}$ is crucial for arriving at $Opt(ARC) = Opt(RC)$. □

Example 8 **(Assumption iv)** As a counterexample for cases in which Assumption iv is not satisfied, we can use the problem in Example 7 with $\mathcal{Z} = [-1, 2]$ and $\mathcal{Y} = \{-1, 1\}$. Then, $Opt(ARC) = 0 < Opt(RC) = 1$. □

Example 9 **(Assumption vi)** Consider the problem

$$\begin{aligned} \min \quad & -y_1 - y_2 \\ s.t. \quad & \zeta^2 + (1-\zeta)y_1 + (1+\zeta)y_2 \leq 3, \\ & |y_i| \leq 3, \quad i = 1, 2, \end{aligned} \tag{36}$$

where $\zeta \in [-1, 1]$ is an uncertain parameter and $y = (y_1, y_2)$ is an adjustable variable. It is clear that (36) is not concave in ζ, but convex in y_1 and y_2. Also, $\mathcal{Z} = [-1, 1]$ and $\mathcal{Y} = \{(y_1, y_2) : \ |y_i| \leq 3, \ i = 1, 2\}$ are compact and convex and the uncertainty is constraint-wise. The (RC) corresponding to (36) is as follows:

$$\begin{aligned} \min \quad & -y_1 - y_2 \\ s.t. \quad & \max_{\zeta \in [-1,1]} \left[\zeta^2 + (1-\zeta)y_1 + (1+\zeta)y_2\right] \leq 3, \\ & |y_i| \leq 3 \quad i = 1, 2. \end{aligned}$$

Due to the fact that the maximum value of a convex function over a convex set is attained at one of the extreme points [1, Theorem 3.4.7], (RC) is equivalent to the following problem whose optimal objective value is -2:

$$\begin{aligned} \min \quad & -y_1 - y_2 \\ s.t. \quad & y_1 \leq 1, \\ & y_2 \leq 1, \\ & |y_i| \leq 3, \quad i = 1, 2. \end{aligned}$$

To get an upper bound for $Opt(ARC)$, we choose $y_1(\zeta) = \frac{3}{2}(1+\zeta)$ and $y_2(\zeta) = \frac{3}{2}(1-\zeta)$ as a decision rule, and it is easy to check the feasibility of $(y_1(\zeta), y_2(\zeta))$. Hence, an upper bound for $Opt(ARC)$ is

$$\max_{\zeta \in [-1,1]} -y_1(\zeta) - y_2(\zeta) \ = \ \max_{\zeta \in [-1,1]} -3 \ = \ -3.$$

So, $Opt(ARC) \leq -3 < -2 = Opt(RC)$. □

Example 10 **(Assumption vii)** Consider the problem

$$\begin{aligned} \min \ & t \\ s.t. \ & |y_1| \leq t, \\ & |y_2| \leq t, \\ & -(y_1 - \zeta_1)^2 - (y_2 - \zeta_1)^2 \leq -4 - 2\zeta_1^2, \\ & -(y_1 - \zeta_2)^2 - (y_2 - \zeta_2)^2 \leq -4 - 2\zeta_2^2, \\ & |y_i| \leq 5, \quad i = 1, 2, \end{aligned} \tag{37}$$

where $\zeta_1 \in [-1, 2]$ and $\zeta_2 \in [-2, 1]$ are the uncertain parameters and $y = (y_1, y_2)$ is an adjustable variable. It is easy to check that (37) is concave (and, more precisely, it is linear) in the uncertain parameter ζ, and the uncertainty is constraint-wise. Also, $\mathcal{Z} = [-1, 2] \times [-2, 1]$ and $\mathcal{Y} = [-5, 5] \times [-5, 5]$ are convex and compact. However, the problem is not convex in the adjustable variable $y = (y_1, y_2)$. The (RC) corresponding to (37) is equivalent to

$$\begin{aligned} \min \quad & \|y\|_\infty \\ s.t. \quad & (y_1 + 1)^2 + (y_2 + 1)^2 \geq 6, \\ & (y_1 - 2)^2 + (y_2 - 2)^2 \geq 12, \\ & (y_1 - 2)^2 + (y_2 + 2)^2 \geq 12, \\ & (y_1 + 1)^2 + (y_2 - 1)^2 \geq 6, \\ & |y_i| \leq 5, \quad i = 1, 2. \end{aligned} \tag{38}$$

It is easy to verify that the optimal solution is $y_1 = -\frac{2+\sqrt{14}}{5} \approx -1.15$ and $y_2 = \frac{5+\sqrt{127+6\sqrt{14}}}{5} \approx 3.44$, with the approximated objective value 3.44 for the problem. We choose

$$y_1(\zeta) = \begin{cases} -1.7, & \zeta_2 \leq 0.3 \\ 1.6, & o.w. \end{cases}, \quad y_2(\zeta) = \begin{cases} 2.2, & \zeta_2 \leq 0.3 \\ -1.6, & o.w. \end{cases}$$

as a decision rule to find an upper bound for $Opt(ARC)$. The feasibility of the decision rule can be easily checked, and it implies that $Opt(ARC) \leq 2.2 < 3.44 \approx Opt(RC)$. □
